# We Should Not Use Randomization Procedures to Allocate Scarce Life-Saving Resources

**DOI:** 10.1093/phe/phab025

**Published:** 2021-11-26

**Authors:** Roberto Fumagalli

**Affiliations:** King’s College London, UK, London School of Economics, UK and University of Pennsylvania, USA

## Abstract

In the recent literature across philosophy, medicine and public health policy, many influential arguments have been put forward to support the use of randomization procedures (RAND) to allocate scarce life-saving resources (SLSR). In this paper, I provide a systematic categorization and a critical evaluation of these arguments. I shall argue that those arguments justify using RAND to allocate SLSR in fewer cases than their proponents maintain and that the relevant decision-makers should typically allocate SLSR directly to the individuals with the strongest claims to these resources rather than use RAND to allocate such resources.

## Introduction

During the COVID-19 pandemic emergency, dramatic increases in the demand for intensive care units have occurred worldwide (e.g. Antommaria *et al.*, 2020; White and Lo, 2020). In several countries, the number of patients needing intensive care exceeded the number of available intensive care units, forcing medical personnel to make thorny triage decisions (e.g. Cook, 2020; Truog *et al.*, 2020). Most of the criteria proposed for allocating scarce intensive care units involve a combination of medical need, various clinical factors (e.g. presence and severity of other medical conditions, expected speed of recovery) and some *randomization procedures* (henceforth, RAND; e.g. British Medical Association, 2020a,b; Chisholm, 2020; Emanuel *et al.*, 2020). The use of RAND has been advocated to allocate many types of *scarce life-saving resources* (henceforth, SLSR) besides intensive care units (e.g. urgent organs for transplant, vaccines, organ replacement therapy). In the recent literature across philosophy, medicine and public health policy, many influential arguments have been put forward to support the use of RAND to allocate SLSR (e.g. Sher, 1980; Broome, 1984a,b, 1991a, b; Elster, 1988; Waring, 2004; Saunders, 2009). The idea is that in cases where dividing the available SLSR among the individuals in need is impossible or significantly reduces SLSR’s expected benefits, the relevant decision-makers (e.g. individual doctors, ethical committees, governmental organizations) should allocate SLSR on the basis of RAND (e.g. Glover, 1977; Kornhauser and Sager, 1988; Goodwin, 1992; Hooker, 2005; Stone, 2007, 2011).

In this paper, I provide a systematic categorization and a critical evaluation of the most influential arguments that have been put forward to support the use of RAND to allocate SLSR. I shall examine in turn: the argument from *convenience*; the argument from *tie-breaking*; the argument from *equal chances*; the argument from fairness; the argument from *incommensurability*; and the argument from *unjust discrimination*. I shall argue that these arguments justify using RAND to allocate SLSR in fewer cases than their proponents maintain and that the relevant decision-makers should typically allocate SLSR *directly* to the individuals with the strongest claims to SLSR, i.e. use what I call *direct allocation procedures* (henceforth, DIR) rather than RAND to allocate SLSR. To be sure, my claim is not that *all* DIR are more justified allocation procedures than RAND. For in many allocation problems, one may identify several DIR that are less justified than RAND (e.g. think of DIR grounded on racist criteria). Rather, my point is that in most allocations of SLSR, one may identify at least *some* DIR that are more justified than RAND and should therefore use such DIR to allocate the available SLSR. If correct, my claim that the relevant decision-makers should typically use DIR (rather than RAND) to allocate SLSR has widespread implications not just for the individuals involved in these allocations, but also for public policy and clinical practice across the biomedical/the health sciences. For as illustrated in the coming sections, many proposals for allocating SLSR in public policy and the biomedical/the health sciences rest on presuppositions concerning the relative merits of DIR and RAND.^1^

Before proceeding, three preliminary remarks are in order. First, I speak of *SLSR* broadly to encompass a wide range of medical treatments and therapies that are critical to patients’ survival (e.g. intensive care units, urgent organs for transplant, vaccines, organ replacement therapy). Moreover, I use the term *RAND* to indicate allocation procedures that are designed to yield specific distributions of the chances that the involved individuals have of receiving the available SLSR (e.g. tossing a coin, rolling a die, picking balls from urns). Some RAND (henceforth, *weighted* RAND) apportion each individual’s chance of receiving the available SLSR to the strength of this individual’s claim to such SLSR. Other RAND (henceforth, *unweighted* RAND) give to each of the involved individuals an equal chance of receiving the available SLSR irrespective of how strong each individual’s claim to such SLSR is. Yet, by design, neither weighted nor unweighted RAND allow the relevant decision-makers to directly determine which individuals will receive the available SLSR before such RAND are implemented (e.g. Stone, 2011: ch. 2; also ‘Argument from Unjust Discrimination' on the ‘sanitizing’ function of lotteries).^2^

Second, I use the term *DIR* to indicate allocation procedures that give SLSR *directly* to the individuals with the strongest claims to these SLSR rather than allocate such SLSR on the basis of RAND (e.g. allocation procedures based on medical need and clinical factors such as presence and severity of other medical conditions and expected speed of recovery). Designing and implementing DIR require the relevant decision-makers to rank the involved individuals according to the strength of their claims to the available SLSR and allocate these SLSR to the individuals with the strongest claims to such SLSR. RAND and DIR may occasionally support similar (or even identical) allocations of the available SLSR. Even so, profound differences remain in the *justificatory principles* which ground the use of RAND and DIR, respectively. For on DIR, the strength of individuals’ claims to the available SLSR directly determines not only what *chances* these individuals have of receiving these SLSR, but also which *individuals* will receive such SLSR (e.g. Broome, 1991a: 98; Hooker, 2005: 348–349). Below I argue that the relevant decision-makers should typically use DIR (rather than RAND) to allocate SLSR, but I do not take a position about the issue of which DIR should be used to allocate specific SLSR. In particular, my call for DIR is compatible with the pluralist view that which DIR should be used in a given allocation problem may depend on various contextual features of such problem, including what type of SLSR is allocated (e.g. urgent organs for transplant versus intensive care units), how much the involved individuals’ claims differ in strength, and how stringent the scarcity constraints faced by the relevant decision-makers are.^3^

And third, there are many clinical and non-clinical grounds (e.g. medical need and conditions, age, desert, rights, willingness to pay, waiting time) on which one might ascribe to individuals *claims* to SLSR (e.g. Basson, 1979; Broome, 1991a; Elhauge, 1994; Schmidt, 1998; Harris and Erin, 2002; Persad *et al.*, 2009; Savulescu *et al.*, 2019; John and Millum, 2020; Wilkinson *et al*., 2020). Still, not all the reasons that an individual may have for receiving SLSR ground claims to such SLSR (e.g. the mere fact that an individual thinks that she would benefit from receiving some SLSR does not *per se* ground a claim to such SLSR). I am not concerned here with demarcating which particular reasons ground claims to SLSR and what factors determine the strength of individuals’ claims to SLSR in specific allocation problems (e.g. Broome, 2004; Childress and Beauchamp, 2009; Voorhoeve, 2014; Sharadin, 2016; Emanuel *et al.*, 2020, for recent discussion). For my evaluation, it suffices to note that if an individual has a claim to some SLSR, then there is a reason to think that the individual ought to receive this SLSR, but there may be overarching reasons not to give such SLSR to the individual (e.g. Broome, 1994; Stone, 2011: ch. 4; Tomlin, 2012; Kirkpatrick and Eastwood, 2015, on cases where other individuals have stronger claims to the available SLSR).^4^

## Argument from Convenience

The *argument from convenience* holds that the relevant decision-makers should use RAND (rather than DIR) to allocate SLSR on the alleged ground that designing and implementing RAND is typically less costly and time-consuming than designing and implementing DIR (e.g. Persad *et al.*, 2009; also Duxbury, 1999: 72, claiming that it is ‘appropriate and beneficial to resort to [RAND] where a cost-effective method of decision-making is required’). The idea is that acquiring the information required to establish which candidates have the strongest claims to SLSR is ‘expensive and time consuming’ and that RAND avoid ‘the costs […] of deliberate selection’ (Broome, 1991a: 88; also Elster, 1988: 169, claiming that RAND are ‘rationally prescribed […] because of their simplicity and universal applicability’).

There are at least two reasons to doubt that the argument from convenience justifies using RAND (rather than DIR) to allocate SLSR. First, convenience is not *the only* factor that bears on the justifiability of the available procedures for allocating SLSR. In particular, time and resources are rarely *so* limited that they prevent the relevant decision-makers from being able to assess how the available allocation procedures fare in terms of factors other than convenience (e.g. fairness). To be sure, one can envision situations where convenience considerations have paramount importance (e.g. think of situations of unforeseen emergency where the relevant decision-makers have limited time and resources to assess different allocation procedures). Still, even in those situations, the relevant decision-makers typically have sufficient time and resources to examine at least *some* of the factors besides convenience that bear on the justifiability of the available allocation procedures (e.g. medical need and various clinical factors). And considerations of these factors often enable the relevant decision-makers to make some informed ordinal judgements regarding the strength of the involved individuals’ claims to SLSR (e.g. Antommaria *et al.*, 2020; Chisholm, 2020, on doctors’ widespread reliance on considerations of medical need and various clinical factors to allocate intensive care units during the first wave of the COVID-19 pandemic emergency).

And second, even if convenience was the main factor that bears on the justifiability of the available procedures for allocating SLSR, convenience considerations *rarely* (if ever) justify using RAND to allocate SLSR. For there are *plenty* of different (weighted and unweighted) RAND one could use in a given allocation problem. And establishing *which* RAND are justifiably adopted in a given allocation problem (e.g. weighted versus unweighted RAND, which weighted or unweighted RAND) requires the relevant decision-makers to determine how much the involved individuals’ claims to the available SLSR differ in strength and how exactly such differences bear on the justifiability of the proposed RAND (e.g. Den Hartogh, 2004: 17). Regrettably, determining how much the involved individuals’ claims to the available SLSR differ in strength and how exactly such differences bear on the justifiability of the proposed RAND requires time and resources. And for all the argument from convenience shows, RAND may commonly be as costly as DIR in terms of time and resources. In fact, weighted RAND will likely be more costly than DIR in all those cases where identifying the individuals with the strongest claims to the available SLSR is easier than allocating to each of the involved individuals chances that are proportional to the strength of each of these individuals’ claims to such SLSR (e.g. Broome, 1984b; John and Millum, 2020).

A proponent of the argument from convenience may object that when allocating SLSR, it is rarely convenient to attempt to identify which individuals have the strongest claims to such SLSR because the *difference in the strength* of distinct individuals’ claims to SLSR is typically ‘*small* compared with the *cost* of acquiring the additional information’ (Elster, 1988: 120, italics added; also Stone, 2014: 198, on putative cases where the relevant decision-makers could differentiate the strength of individuals’ claims ‘given further information, but obtaining that information would be too difficult or costly to justify making the effort’). The objection correctly notes that identifying which individuals have the strongest claims to SLSR may occasionally involve expensive and time-consuming evaluations. However, it is dubious that the difference in the strength of distinct individuals’ claims to SLSR is typically ‘small compared with the cost of acquiring the additional information’. For allocations of SLSR have crucial life-and-death implications whose normative relevance typically trumps the cost of acquiring the information required to identify which individuals have the strongest claims to SLSR (e.g. Calabresi and Bobbitt, 1978; Broome, 1984b; Kilner, 1990; Iapichino *et al.*, 2010). In this respect, it would be of limited import to allege that ‘time pressure and limited information [may make RAND] preferable to trying to make finer-grained [evaluations] within a group of […] similar patients’ (Emanuel *et al.*, 2020: 2053). For the relevant decision-makers need time and resources to establish whether the involved individuals’ claims to SLSR are ‘similar’ in strength and how ‘similar’ in strength they are. And as I argue in the coming sections, the proponents of RAND are rarely able to demonstrate that individuals’ claims to SLSR are sufficiently similar in strength to justify using RAND (rather than DIR) to allocate SLSR.

A proponent of the argument from convenience may further object that when allocating SLSR, RAND *relieve* the relevant decision-makers of ‘the *responsibility* of deciding who is to live and who to die [and of the associated] *emotional burden*’ (Broome, 1991a: 88, italics added; also Glover, 1977: 219). However, using RAND to allocate SLSR does not generally alleviate the relevant decision-makers’ emotional burden. For their decisions ‘will make all the difference between life and death anyway’ (Henning, 2015: 194) irrespective of whether they use RAND or DIR. Moreover, even if using RAND generally alleviated the relevant decision-makers’ emotional burden, the psychological benefits that using RAND (rather than DIR) putatively yields to the relevant decision-makers (while normatively relevant) would rarely trump the normative relevance of the differences in strength of different individuals’ claims to SLSR and these differences’ life-and-death implications. More generally, the point remains that the availability of RAND does not *per se* relieve the relevant decision-makers of the responsibility of assessing the strength of different individuals’ claims to the available SLSR. For even in those cases where they decide to use RAND, the relevant decision-makers are responsible for their decision to allocate SLSR on the basis of RAND rather than DIR. And in such cases, it is up to the relevant decision-makers to explicate on what grounds exactly individuals’ living or dying should be left to chance ‘when in so many other areas of human life we believe that we have an obligation to [allocate benefits and burdens on the basis of DIR]’ (Harris, 1975: 83; also Wolfle, 1970: 1201; Belliotti, 1980: 255).^5^

## Argument from Tie-Breaking

The *argument from tie-breaking* holds that the relevant decision-makers should use RAND (rather than DIR) to allocate SLSR on the alleged ground that RAND ‘are good tie breakers’, i.e. they are an effective ‘means of getting the decision made [when the claims] of different candidates are exactly balanced’ (Broome, 1991a: 89; also Elster, 1988: 107–113; Goodwin, 1992). The idea is that in all situations where ‘two or more people have equal claims’ to SLSR, unweighted RAND are ‘the morally preferable way of allocating [SLSR]’ (Sher, 1980: 203; also Kornhauser and Sager, 1988; Stone, 2007, 2009). For in those situations, using allocation procedures other than unweighted RAND ‘is either to say that [the involved people’s] claims were not equal [or] to unjustly favour one of the equal claims over the other on the basis of irrelevant differences’ (Saunders, 2008: 362).

There are at least two reasons to doubt that the argument from tie-breaking justifies using RAND (rather than DIR) to allocate SLSR. First, the relevant decision-makers are not always able to *establish* whether different individuals’ claims to SLSR are *equally strong*. For the strength of individuals’ claims to SLSR depends on multiple factors, and it is frequently difficult to assess reliably the overall impact of such factors on the strength of individuals’ claims to SLSR (e.g. White *et al.*, 2009; Emanuel *et al.*, 2020; also ‘Argument from Incommensurability' on how this difficulty hampers the justifiability of various DIR). And second, in cases where the relevant decision-makers are able to reliably assess the strength of individuals’ claims to SLSR, different individuals’ claims *rarely* have *exactly* equal strength (e.g. Kirkpatrick and Eastwood, 2015: 87, claiming that situations where ‘the claims of two candidates are exactly equal and we know that their claims are exactly equal [are] incredibly rare’; also Broome, 1991a; Den Hartogh, 2004; Voorhoeve, 2014, on the frequent cases where individuals significantly differ in terms of medical need or likelihood to benefit from the available SLSR). In this respect, it would be of limited import to allege that in ‘matters of life and death […] everyone has a claim to life’ (Broome, 1991a: 99; also Kilner, 1981). For the alleged fact that all the involved individuals have *a* claim to *life* falls short of implying that such individuals have *equally strong* claims to *SLSR* (e.g. Fumagalli, 2018; also Broome, 1984b: 40, holding that ‘when choosing between people it will never in practice happen that all the considerations in favor of [each] candidate will exactly balance’). These considerations, in turn, cast doubt on the argument from tie-breaking’s potential to ground a wide-ranging case in favour of RAND. As Sher puts it, RAND’s role in ‘breaking a tie […] would be insignificant [since] it will hardly ever happen in practice that [claims] balance exactly. And if ever they do, the slightest change in one of them would mean they were no longer balanced’ (1980: 203; also Den Hartogh, 2004: 18).^6^

A proponent of the argument from tie-breaking may object that if one chooses to allocate SLSR directly to some individuals (rather than others), then one is treating some individuals’ lives as *more worthy* than others (e.g. Saunders, 2008: 362). Still, one may consistently hold that different individuals’ *lives* are equally worthy, yet maintain that these individuals’ *claims* to specific SLSR differ in strength (e.g. Broome, 1994: 36–37). In this respect, it would be implausible to hold that since individuals’ lives are equally worthy, individuals should be given equal chances of receiving SLSR even when their claims significantly differ in strength (e.g. Waring, 2004: ch. 1). For the alleged fact that individuals’ lives are equally worthy falls short of implying that the relevant decision-makers can justifiably disregard major differences in the strength of distinct individuals’ claims to SLSR (e.g. think of major differences in medical need and expected benefits from treatment). And whenever the relevant decision-makers can identify major differences in the strength of distinct individuals’ claims to SLSR, ‘selecting randomly is [neither] a way of refusing to judge between people […] nor is it a way of affirming people’s equal worth as human beings’ (Broome, 1984b: 52).

A proponent of the argument from tie-breaking may further object that, in concrete allocation problems, decision-makers often reach a point where they are *unable* to reliably identify any major differences in the strength of distinct individuals’ claims to SLSR (e.g. Rescher, 1969; White *et al.*, 2009, on two-stage procedures where one first uses DIR to narrow down the set of potential candidates and then singles out the actual patients within this group by means of RAND). The idea is that one can justifiably use RAND as tie-breakers when allocating SLSR among individuals who are ‘tied within the limits of comparability’ (Broome, 1991a: 101; also Stone, 2013: 591, holding that in many situations, individuals’ claims are ‘so similar’ that the relevant decision-makers ‘are more likely to [wrongfully distinguish] among candidates with equal claims [than they are to reliably distinguish] among candidates with unequal claims’). This objection correctly notes that in some situations, the relevant decision-makers are unable to reliably identify major differences in the strength of distinct individuals’ claims to SLSR (e.g. Savulescu, 1998). Still, in many real-life allocation problems, the relevant decision-makers can reliably identify differences that are significant enough to break putative ties between individuals (e.g. the coming sections on major differences in medical need and various clinical factors; also Fumagalli, 2020, on decision-makers’ ability to identify morally significant differences across several policy contexts). Whenever this is the case, carefully designed and implemented DIR are more likely than RAND to allocate SLSR to individuals having comparatively stronger claims to such SLSR (e.g. ‘Argument from Fairness'). Moreover, even in cases where the relevant decision-makers are unable to identify differences that are significant enough to break putative ties between individuals, it is questionable whether RAND are uniquely justified to break those ties. For in such cases, many allocation procedures that do not involve any kind of RAND may be justifiably used to break ties (e.g. Ullmann-Margalit and Morgenbesser, 1977, on various so-called picking procedures). In this respect, it is telling that leading proponents of RAND concede that when allocating SLSR, ‘it is doubtful that [RAND are] going to be the best way of breaking the tie’ (Broome, 1991a: 101).

A proponent of the argument from tie-breaking may further object that RAND ‘become live options *only after* some effort to [filter] out options has taken place’ (Stone, 2011: 149, italics added; also Elster, 1989: 67, claiming that ‘I know of no instance of social lotteries without some preselection […] on the basis of need, merit, and the like’). This objection correctly notes that RAND are rarely implemented without *some* preliminary screening of the involved individuals’ claims to the available SLSR. Still, the objection fails to ground a wide-ranging case in favour of RAND unless it is supplemented with plausible and detailed criteria to establish *what level* of preliminary screening is adequate. In this respect, it would be of limited import to contend that RAND are justifiably used ‘after the “*right*” amount of work has been done by reasons’ and that the relevant decision-makers should use RAND whenever ‘the dangers posed by *bad* reasons outweigh the benefits offered by *good* reasons’ (Stone, 2011: 150, italics added). For these contentions do not specify what the ‘right’ amount of work consists in, and leave unclear how the relevant decision-makers are supposed to demarcate ‘good’ and ‘bad’ reasons and how they should resolve disagreements about this demarcation issue. Moreover, DIR are not the only allocation procedures that face the risk of grounding allocations of SLSR on reasons that do not bear on the strength of individuals’ claims to SLSR. For weighted RAND also face such risk (e.g. Stone, 2013). And unweighted RAND face the opposite—and often more troublesome—risk of failing to track reasons that decisively bear on the strength of individuals’ claims to SLSR (e.g. Teira, 2013a).^7^

## Argument from Equal Chances

The *argument from equal chances* holds that the relevant decision-makers should use RAND (rather than DIR) to allocate SLSR on the alleged ground that when different individuals have equally strong claims to SLSR and dividing SLSR is not possible (or substantially decreases their expected benefits), ‘justice demands that each person receive an “equal chance” at getting [SLSR]’ (Stone, 2007: 281; also Stone, 2011: 17). The idea is that RAND have ‘the unique capacity to divide a good probabilistically’ (Kornhauser and Sager, 1988: 491) and enable the relevant decision-makers to substitute equality of outcomes with equality of chance, which is a ‘second-best equity’ (Duxbury, 1999: 72–73) and a ‘surrogate satisfaction’ of the individuals’ claims to SLSR (Broome, 1991a: 98; also Elster, 1988: 128; Saunders, 2008: 359).^8^

There are at least two reasons to doubt that the argument from equal chances justifies using RAND (rather than DIR) to allocate SLSR. First, as noted in ‘Argument from Tie-Breaking', the proponents of RAND are *rarely* able to establish that different individuals’ claims to SLSR have equal strength. This, in turn, casts doubt on the argument from equal chances’ potential to ground a wide-ranging case in favour of RAND. In particular, it indicates that the allocative decision to give each of the involved individuals the same chance of receiving the available SLSR ‘requires justifying as much as any other’ allocative decision (Broome, 1984b: 54). And second, whenever the relevant decision-makers have reason to think that the involved individuals’ claims to the available SLSR significantly *differ* in strength, it is dubious that giving each of the involved individuals the same chance of receiving this SLSR has the *normative relevance* required to justify using RAND to allocate such SLSR. In fact, it is hard to see in what sense ‘chances of satisfaction [would constitute] *surrogate satisfaction*, so that giving people equal chances would constitute a way to treat [people’s] claims fairly’ (Henning, 2015: 177, italics added; also Lazenby, 2014: 337, holding that giving one some chance of receiving a good does not *per se* provide her with any ‘satisfaction of her claim […] commensurate with satisfaction in outcome’).

A proponent of the argument from equal chances may object that ‘the provision of […] epistemically equal positive chances of an indivisible, life-saving resource to those with equal claims […] ensures the equal distribution of something that it is *rational* for different individuals to *prudentially value* equally’ (Otsuka, 2021, italics added; also Parfit, 2003: 376–378). This objection correctly notes that the involved individuals might regard their chances of receiving SLSR as intrinsically (rather than just instrumentally) valuable (e.g. Goldschmidt and Nissan-Rozen, 2021). Still, the chances given by RAND do not directly benefit the involved individuals in a way that makes it prudentially rational for these individuals to value an equal distribution of chances. In particular, it is not generally the case that ‘the chance of a benefit is itself a benefit’ (Saunders, 2008: 367; also Broome, 1984a; Wasserman, 1996). In fact, it would be mistaken to generally regard the chances given by RAND as an *additional* good to be added to the expected benefit of receiving SLSR (e.g. Henning, 2015: 174, holding that it would ‘be double counting to regard the chances provided by [RAND] as additional goods’).^9^

A proponent of the argument from equal chances may further object that ‘though the chance of a benefit is not itself a benefit […] we should not ignore such chance’ (Parfit, 2003: 377). In particular, she may maintain that when allocating SLSR, differences in the strength of different individuals’ claims to the available SLSR are often of *minor normative relevance* compared to the importance of giving each of the involved individuals equal chances of receiving such SLSR (e.g. Harris, 1999; Segev, 2005). The idea is that the relevant decision-makers should use unweighted RAND to allocate SLSR on the alleged ground that the importance of giving each of the involved individuals equal chances trumps the normative relevance of the differences in the strength of these individuals’ claims. However, when allocating SLSR, the importance of giving each of the involved individuals equal chances rarely trumps the normative relevance of the differences in the strength of these individuals’ claims. For as noted in the previous sections, there frequently are major differences in the strength of distinct individuals’ claims to SLSR, and allocations of SLSR often have life-and-death implications whose normative relevance trumps the importance of giving each of the involved individuals equal chances. To put it differently, when the relevant decision-makers can identify major differences in the strength of distinct individuals’ claims to SLSR, the reason to prefer individuals with comparatively stronger claims outweighs the importance of giving each individual an equal chance (e.g. Savulescu, 1998; Stein, 2002). In this respect, it would be of limited import for a proponent of the argument from equal chances to object that if individuals’ claims significantly differ in strength, then the relevant decision-makers can track such difference by relying on weighted (rather than unweighted) RAND (e.g. Broome, 1991a: 98). For weighted RAND do not give each of the involved individuals an equal chance of receiving the available SLSR and face severe difficulties when it comes to apportioning each individual’s chance of receiving the available SLSR to the strength of each individual’s claim to such SLSR (e.g. Kirkpatrick and Eastwood, 2015: 82, holding that ‘it is almost impossible to calculate the weights of the lotteries in accordance with the [strength of each individual’s claim]’; also the previous sections).^10^

## Argument from Fairness

The *argument from fairness* holds that the relevant decision-makers should use RAND (rather than DIR) to allocate SLSR on the alleged ground that RAND are typically fairer than DIR and are ‘sometimes […] the fairest way of distributing a good’ (Broome, 1991a: 87–88; also Rowe, 2021). The idea is that RAND yield a fairness gain compared to DIR because fairness ‘requires that each candidate’s claim should be satisfied in proportion to its strength’ and RAND satisfy this requirement better than DIR (Broome, 1994: 38; also Broome, 1984b: 45, 1991a: 95). To be sure, the argument grants that RAND involve a fairness loss compared to DIR in that they are less likely than DIR to allocate SLSR to individuals with comparatively stronger claims to SLSR (e.g. Broome, 1984b). Still, the argument holds that this fairness loss must ‘be weighed against [RAND’s] contribution to fairness’ and that ‘if claims are close to equality, holding [RAND] will be fairer than not’ (Broome, 1991a: 99; also Stone, 2011: 88, holding that when the involved individuals have ‘equal claims […] it is impossible to imagine anything fairer’ than RAND). Hence, the argument goes, when allocating SLSR, RAND are typically justified because in ‘matters of life and death, fairness is particularly important [and] everyone has a claim to life’ (Broome, 1991a: 90 and 99). That is to say, when claims are equal, one should use unweighted RAND, and when claims are not equal, one should use weighted RAND, with heavier weights assigned to the individuals with stronger claims to the available SLSR (e.g. Broome, 1991a; White and Angus, 2020; Rowe, 2021).

There are at least two reasons to doubt that the argument from fairness justifies using RAND (rather than DIR) to allocate SLSR. First, as the argument acknowledges, fairness is not *the only* factor which bears on the justifiability of using RAND (rather than DIR) to allocate SLSR (e.g. Broome, 1991a: 99, holding that the justifiability of using RAND will depend on ‘how important fairness is’ compared to the issue whether ‘the candidates’ claims are equal’). And for all the argument shows, fairness considerations may frequently fail to trump other factors when assessing the justifiability of different procedures for allocating SLSR (e.g. Schmidt, 1994; Nissan-Rozen, 2019; Savulescu *et al.*, 2020, on various cases where major social welfare gains override the demands of fairness). And second, the argument does not show that allocating SLSR on the basis of RAND is *generally* fairer than allocating SLSR on the basis of DIR. This point can be explicated as follows. Several conceptions of fairness have been advocated in the recent literature on the allocation of scarce resources (e.g. Fleurbaey, 2008; Otsuka and Voorhoeve, 2009; Fleurbaey and Maniquet, 2011). Few of the proffered conceptions endorse the argument from fairness’ presupposition that fairness requires that each candidate’s claim should be satisfied in proportion to its strength (e.g. Saunders, 2010; Tomlin, 2012; Voorhoeve and Fleurbaey, 2012). In fact, various conceptions hold that when individuals’ claims to SLSR *significantly* differ in strength, it would be *unfair* to allocate SLSR using RAND (e.g. Voorhoeve, 2014; also Stone, 2011: 150, holding that it would be unfair to ‘toss […] a coin before deciding whether [to cure] a moderately sick or critically ill person’). For in presence of significant differences in the strength of individuals’ claims, the fairness gain that RAND putatively yield compared to DIR does not offset the fairness loss occurring whenever individuals with comparatively weaker claims receive the available SLSR. As Hooker puts it, ‘suppose your claims to some indivisible good are very much [stronger] than mine. Is there any unfairness in your getting the indivisible good rather than my getting it? […] Given that your claim is so much stronger than mine […] letting the stronger claim win seems completely fair’ (2005: 349).^11^

A proponent of the argument from fairness may object that the argument justifies using RAND (rather than DIR) to allocate SLSR on the alleged ground that *any* change in the strength of the involved individuals’ claims to SLSR ‘warrant[s] a *similarly-sized* change to what *fairness* requires’ (Rowe, 2021: 1, italics added). I am not persuaded that any change in the strength of the involved individuals’ claims to SLSR warrants ‘a similarly-sized change to what fairness requires’. For the fairness loss involved in using RAND (rather than DIR) increases as the difference in the strength of the involved individuals’ claims increases. And for all the argument from fairness shows, RAND may be overall fair only when the involved individuals have claims of the same strength (e.g. Lazenby, 2014: 344; also Saunders, 2010; Voorhoeve, 2014; Kirkpatrick and Eastwood, 2015, on cases where the difference in the strength of distinct individuals’ claims to SLSR is so large that it seems rather unfair to use RAND to decide which individuals receive such SLSR). This, in turn, significantly constrains the argument from fairness’ potential to ground a wide-ranging case in favour of RAND. For as argued in the previous sections, individuals’ claims rarely have the same strength . Moreover, even assuming that any change in the strength of the involved individuals’ claims to SLSR warranted ‘a similarly-sized change to what fairness requires’, this assumption would not *per se* license the conclusion that any change in the strength of the involved individuals’ claims to SLSR warrants a similarly-sized change in the *chance* that each of the involved individuals should have of receiving SLSR.

A proponent of the argument from fairness may further object that the argument ‘can be extended’ from cases where candidates have ‘*exactly* equal claims’ to SLSR to cases where they have ‘*roughly* equal claims’ to SLSR, thereby vindicating the use of RAND in ‘a much wider domain’ (Broome, 1984b: 48, italics added; also Rowe, 2021, on cases where decision-makers are unable to reliably identify major differences in the strength of individuals’ claims). The idea is that the argument from fairness justifies using RAND (rather than DIR) to allocate SLSR *unless* the involved individuals’ claims *significantly* differ in strength and enables the relevant decision-makers to identify *clear cases* where using RAND is justified. To illustrate this objection, consider the following scenario (e.g. Piller, 2017: 230, for a similar illustration). Assume, for the sake of argument, that we can reliably measure the strength of individuals’ claims to SLSR on some cardinal scale. Compare then a *large-difference* case where a 99 per cent to 1 per cent distribution of the chances of receiving SLSR reflects the strength of two individuals’ claims to SLSR, with a *small-difference* case where a 51 per cent to 49 per cent distribution of the chances of receiving SLSR reflects the strength of two individuals’ claims to SLSR. The argument from fairness does not recommend using RAND in the large-difference case since the fairness loss which occurs when using DIR that reduce the chance of the individual with the rather weak claim from 1 per cent to 0 per cent is negligible. Conversely, the argument from fairness recommends using weighted RAND in the small-difference case on the alleged ground that using DIR that reduce the chance of the individual with the slightly weaker claim from 49 per cent to 0 per cent is less fair than using RAND that give such individual a 49 per cent chance of receiving SLSR. The idea is that in the small-difference case, allocating SLSR directly to the individual with the slightly stronger claim would be unfair because the individual with the slightly weaker claim ‘is entitled to treatment only slightly less good’ than the individual with the slightly stronger claim rather than a 0 per cent chance of receiving SLSR (Broome, 1984b: 48).

This objection invites two rejoinders. First, the mere fact that DIR give the individual who would otherwise have a 49 per cent chance of receiving SLSR a 0 per cent chance of receiving SLSR does not *per se* make DIR unfair. In fact, the argument from fairness presupposes (rather than shows) that fairness requires to give such individual a 49 per cent chance of receiving SLSR. In particular, the argument fails to undermine conceptions of fairness according to which, when the relevant decision-makers can *reliably* identify differences in the strength of distinct individuals’ claims to SLSR, fairness requires giving SLSR *directly* to the individuals with the strongest claims to SLSR rather than giving all the involved individuals chances proportional to the strength of these individuals’ claims to SLSR. And second, the argument from fairness does not provide plausible and detailed criteria to establish exactly when the difference between stronger and weaker claims can be justifiably regarded as *small enough* that the fairness gain putatively yielded by RAND trumps the unfairness which occurs whenever the individuals with comparatively weaker claims receive SLSR. Hence, the argument does not provide plausible and detailed criteria to establish in what situations RAND are fairer than DIR.^12^

## Argument from Incommensurability

The *argument from incommensurability* holds that the relevant decision-makers should use RAND (rather than DIR) to allocate SLSR on the alleged ground that ‘when comparing candidates for life-saving treatment […] it often seems impossible to weigh the [candidates’ claims to treatment] in a precise way’ (Broome, 1991a: 100; also Glover, 1977: 203–227). The idea is that the strength of different individuals’ claims to SLSR is often incommensurable (e.g. Rescher, 1969; Raz, 1999: ch. 3) and that whenever it is ‘impossible in practice to carry out finely grained comparisons’ of the strength of individuals’ claims to SLSR, the relevant decision-makers should allocate SLSR on the basis of RAND (Elster, 1988: 131; also Broome, 1984b: 50, holding that ‘there are many possible criteria’ for choosing between the involved individuals, and ‘there may not even be any single correct way of weighing up the criteria’).^13^

There are at least three reasons to doubt that the argument from incommensurability justifies using RAND (rather than DIR) to allocate SLSR. First, showing that the strength of different individuals’ claims to SLSR is *incommensurable* requires one to show that reliable comparisons of the strength of such claims are ‘*impossible* […] not just costly or difficult’ (Elster, 1988: 163, italics added). Yet, the proponents of the argument from incommensurability have hitherto failed to show that cases of incommensurability are sufficiently widespread to ground a wide-ranging case in favour of RAND. Second, it is dubious that cases of incommensurability are sufficiently widespread to ground a wide-ranging case in favour of RAND. For as illustrated in the previous sections, the difficulties inherent in assessing the strength of different individuals’ claims to SLSR rarely *prevent* decision-makers from being able to make some informed *ordinal* judgements regarding the strength of different individuals’ claims to SLSR. To give one example, consider decision-makers’ judgements of medical need. When allocating SLSR, it is often difficult to assess how exactly the involved individuals differ in terms of medical need. Still, this difficulty does not prevent the relevant decision-makers from being able to make some informed ordinal judgements regarding individuals’ medical need and how considerations of medical need bear on the strength of those individuals’ claims to SLSR (e.g. Broome, 1984b; also Antommaria *et al.*, 2020; Chisholm, 2020). And third, deciding *which* RAND to use to allocate SLSR (e.g. weighted versus unweighted RAND, which weighted or unweighted RAND) requires the relevant decision-makers to *assess* the strength of different individuals’ claims to SLSR. Hence, pointing to the alleged incommensurability of the strength of different individuals’ claims to SLSR does not *per se* provide a reason to regard RAND as more justified than DIR. As leading proponents of RAND acknowledge, justifying RAND requires one ‘to establish a rank ordering’ among the involved individuals’ claims, and ‘if the claims are incommensurable […] it is unclear precisely what allocative justice demands’ (Stone, 2011: 56).

A proponent of the argument from incommensurability may object that the relevant decision-makers should use RAND (rather than DIR) to allocate SLSR on the alleged ground that ‘when an agent is *unsure* what the morally right thing to do is [then using RAND] is the best one can do, given one’s *moral uncertainty*’ (Nissan-Rozen, 2012: 45–46, italics added; also Rescher, 1969). The idea is that the relevant decision-makers often find it epistemically impossible to identify what procedure it is morally right to use to allocate SLSR and that whenever this is the case, ‘the only rational thing’ for the decision-makers is to use RAND even though the decision-makers have reason to think that there is some other allocation available which is morally better (Nissan-Rozen, 2012: 49). This objection aptly emphasizes the uncertainty that is often involved in identifying what procedure it is morally right to use to allocate SLSR, but does not ground a wide-ranging case in favour of RAND. For as its proponents grant (e.g. Nissan-Rozen, 2012: 45), the objection recommends using RAND only when the relevant decision-makers find it *epistemically impossible* to identify what procedure it is morally right to use to allocate SLSR. And the relevant decision-makers can frequently resolve (or at least alleviate) their moral uncertainty by acquiring *more information* about the allocation problems they face (e.g. which RAND and DIR can be feasibly adopted, what consequences the available allocations may have, and what value different normative theories assign to such allocations).

A proponent of the argument from incommensurability may further object that the relevant decision-makers are *often unable* to resolve their moral uncertainty by acquiring more information about the allocation problems they face (e.g. Hersch and Rowe, 2021, on cases where the relevant decision-makers disagree as to what criteria should be adopted to assess the strength of the involved individuals’ claims). The idea is that there are *limits* to decision-makers’ ability to identify all the reasons that bear on the strength of individuals’ claims to SLSR and that ‘these limits provide reasons for accepting *regular use* of [RAND]’ (Stone, 2013: 579, italics added; also Stone, 2011, 105, holding that many calls for DIR betray ‘extraordinary confidence in what reason can do’). This objection invites two rejoinders. First, one may consistently *advocate* DIR while acknowledging that the relevant decision-makers may be unable to identify all the reasons that bear on the strength of individuals’ claims to SLSR (e.g. ‘Argument from Convenience' on situations of unforeseen emergency). For justifying DIR does not require decision-makers to identify *all* the reasons that bear on the strength of individuals’ claims to SLSR, but only requires them to identify *the main* reasons that bear on the strength of such claims. To be sure, if decision-makers identify only a few of the reasons that bear on the strength of individuals’ claims to SLSR, then it is possible that some unidentified reasons offset the impact that the identified reasons have on the allocation of the available SLSR. Still, if the proponents of RAND are to show that the RAND they advocate are more justified than DIR, then it is up to them to point to unidentified reasons and demonstrate that such reasons do (or at least likely) offset the impact that the identified reasons have on allocation. And second, it is often calls for RAND (rather than calls for DIR) which rest on *unsupported presuppositions* concerning decision-makers’ evaluative abilities. By way of illustration, calls for RAND commonly infer that, if the relevant decision-makers do not happen to find any significant difference in the strength of the involved individuals’ claims to SLSR, then they can justifiably treat these individuals’ claims as if they have equal strength (e.g. Stone, 2009: 395–396). And in many cases, this inference only seems plausible under the presupposition—often left unsupported—that decision-makers’ assessment of the strength of the involved individuals’ claims to SLSR identifies all the main reasons that bear on the strength of these individuals’ claims.^14^

## Argument from Unjust Discrimination

The *argument from unjust discrimination* holds that the relevant decision-makers should use RAND (rather than DIR) to allocate SLSR on the alleged ground that DIR are often unjustly discriminatory (e.g. Stone, 2013; Chisholm, 2020), whereas RAND ‘can [ensure] that bad reasons be kept out of the decision’ (Stone, 2011: vii; also Dowlen, 2008: 15; Saunders, 2008: 359). The idea is that the relevant decision-makers should use RAND (rather than DIR) to allocate SLSR because RAND are less vulnerable than DIR to unjustly discriminatory (e.g. racist) biases and ‘are constitutionally incapable of playing favorites’ (Goodin, 1988: 61; also Kornhauser and Sager, 1988; Stone, 2009, 2011, on the ‘sanitizing’ function of lotteries, which putatively prevents unjustly discriminatory reasons from influencing allocation decisions).^15^

There are at least two reasons to doubt that the argument from unjust discrimination justifies using RAND (rather than DIR) to allocate SLSR. First, a number of factors can make a *significant* difference to the allocation of SLSR across distinct groups of individuals without being *unjustly* discriminatory (e.g. Lippert-Rasmussen, 2013: ch. 1–2). To illustrate this, consider the issue whether SLSR should be allocated on the basis of individuals’ age. Several proposals for allocating SLSR recommend prioritizing the young over the elderly in cases of pandemic emergency where being above a particular age makes individuals significantly less likely to benefit from SLSR (e.g. White and Lo, 2020; British Medical Association, 2020b). Allocating SLSR directly on the basis of individuals’ age is often regarded as unjustly discriminatory (e.g. Popescu and Marcoci, 2020; also Bognar, 2015; Nielsen, 2021, for discussion). Still, the fact that grounding the allocation of SLSR on individuals’ capacity to benefit from SLSR may disadvantage the elderly does not *per se* make such allocation proposals unjustly discriminatory (e.g. Farrelly, 2008; Lazenby, 2011; British Medical Association, 2020a). And second, the fact that discriminatory biases do affect *some* DIR (e.g. Den Hartogh, 2010; Hersch and Rowe, 2021, on cases where first-come first-served and waiting time favour socio-economically privileged individuals) does not cast *general* doubt on the justifiability of DIR. For the proponents of DIR frequently have the means to neutralize (or at least alleviate) the influence of discriminatory biases (e.g.  Antommaria *et al.*, 2020; Truog *et al.*, 2020, on blinding mechanisms to prevent the relevant decision-makers from accessing clinically irrelevant information about individuals’ race and wealth; also British Medical Association, 2020b; White and Lo, 2020, on allocation criteria that explicitly prohibit allocating SLSR on the basis of clinically irrelevant information about individuals’ socio-economic status, ethnicity and sexual orientation). Hence, pointing to some DIR’s vulnerability to discriminatory biases does not *per se* indicate that the relevant decision-makers should allocate SLSR on the basis of RAND.

A proponent of the argument from unjust discrimination may object that the relevant decision-makers should use RAND (rather than DIR) to allocate SLSR on the alleged ground that RAND are *more effective* than DIR in preventing the relevant decision-makers ‘from [engaging in] arbitrary exercise of power’ (Elster, 1988: 166). The idea is that RAND ‘limit the opportunities for corruption and prejudice in decision making [and] guard against partiality and oppression’ (Broome, 1984b: 40; also Stone, 2011: 82). This objection aptly emphasizes the importance of countering the influence of decision-makers’ potential biases and corruption on the design and the implementation of allocation procedures (e.g. Sher, 1980; Teira, 2013b). However, the objection fails to show that RAND are *generally* more effective than DIR in countering the influence of decision-makers’ potential biases and corruption (e.g. think of transparency and accountability concerns pertaining to the decision-makers who design and implement the proffered RAND). In fact, various sources of biases and corruption (e.g. racist attitudes) may demonstrably influence both decision-makers’ calibration of the weights to be adopted in RAND and decision-makers’ judgements as to whether the conditions which putatively justify using RAND (rather than DIR) are satisfied in the first place (e.g. Broome, 1991a: 88; Henning, 2015: 183).^16^

A proponent of RAND may further object that the procedures for allocating specific SLSR are often designed and implemented in contexts facing *structural discrimination* (e.g. think of deep and persistent socio-economic inequalities) and that RAND are generally more effective than DIR in correcting for such discrimination. The idea is that ‘we cannot, nor should we, expect that health-care workers […] redress the social determinants of ill health’ and that in light of existing socio-economic inequalities ‘it seems somewhat disingenuous to pretend that [DIR are not influenced by] the social determinates of health’ (Silva, 2020: 891). Let us assume, for the sake of argument, that the justifiability of the procedures for allocating specific SLSR depends on how effectively these procedures correct for structural discrimination. Even so, it is dubious that RAND are generally more effective than DIR in correcting for structural discrimination. For only DIR and weighted RAND can be calibrated with the specific aim to correct for structural discrimination. And unweighted RAND occasionally tend to exacerbate (rather than correct for) structural discrimination (e.g. John and Millum, 2020, for illustrations targeting iterated applications of unweighted RAND).

## Conclusion

In this paper, I have provided a systematic categorization and a critical evaluation of the most influential arguments put forward to support the use of RAND to allocate SLSR. I have argued that these arguments justify using RAND to allocate SLSR in fewer cases than their proponents maintain and that the relevant decision-makers should typically allocate SLSR directly to the individuals with the strongest claims to SLSR rather than use RAND to allocate SLSR. This result does not *per se* exclude that some further arguments may be articulated which support using RAND to allocate specific SLSR. Still, it makes it pressing for the proponents of RAND to articulate and support those arguments. In the absence of such arguments, the relevant decision-makers should typically allocate SLSR directly to the individuals with the strongest claims to these resources rather than use RAND to allocate such resources.

## Conflict of Interest 

None declared.

## Endnotes

1 RAND are often used to allocate benefits and burdens other than SLSR (e.g. Elster, 1988; Stone, 2013, on the use of lotteries for drafting public servants such as jurors and soldiers and for determining admission to high schools and universities ). My evaluation focuses on the allocation of SLSR (rather than other benefits and burdens) and on allocation problems where decision-makers can save only a given number of individuals among all individuals in need of SLSR. I do not expand on allocation problems where decision-makers can save groups comprising different numbers of individuals since these allocation problems raise additional complexities tangential to my evaluation (e.g. Taurek, 1977; Kamm, 1985; Timmermann, 2004; Otsuka, 2006; Hirose, 2007).

2 A number of factors (e.g. feasibility considerations, the comparative strength of the involved individuals’ claims to SLSR) putatively bear on the justifiability of using weighted (rather than unweighted) RAND to allocate SLSR. I expand on various such factors throughout the paper. Some authors speak of ‘fair’ (rather than ‘unweighted’) RAND to indicate RAND that give to each of the involved individuals an equal chance of receiving the available SLSR (e.g. Sher, 1980: 203). Below I use the term ‘unweighted’ to indicate these RAND since the term ‘fair’ has normative connotations that do not generally characterize such RAND (e.g. ‘Argument from Fairness'). In particular, I use the expression ‘unweighted RAND’ broadly to encompass both RAND that give to the involved individuals objectively equal chances and RAND that give to such individuals epistemically equal chances (e.g. Elster, 1988: 113; Stone, 2007: 280; Saunders, 2008: 363). My critical evaluation of RAND would hold also if one restricted the use of ‘unweighted RAND’ to indicate only RAND that give to the involved individuals objectively equal chances (e.g. Henning, 2015: 175).

3 Below I focus on situations where the relevant decision-makers face scarcity of life-saving resources, without inquiring into the economic, social and political determinants of this scarcity (e.g. Scheunemann and White, 2011; Rowe and Voorhoeve, 2018, on cases where such scarcity results from previous governmental decisions to reduce funding for the health care system). My evaluation of the justifiability of distinct allocation procedures in situations of scarcity in no way exempts the relevant decision-makers from the duty to do all they reasonably can to prevent the scarcity of life-saving resources (e.g. increase investments in the health care system).

4 My evaluation focuses on justificatory (rather than motivating or explanatory) reasons. RAND might be said to allocate SLSR on the basis of reasons in the sense that, according to RAND, an individual receives SLSR because this individual is selected by RAND. These reasons, however, exclusively pertain to the workings of RAND and do not refer to the strength of the individual’s claims (e.g. Sunstein and Ullmann-Margalit, 1999; Stone, 2013).

5 Similar remarks apply to the objection that individuals generally find it easier to accept unfavourable allocations when these allocations result from RAND rather than from DIR (e.g. Rescher, 1969; Bolton *et al.*, 2005; also Stone, 2007: 288, claiming that since RAND are not intended to yield ‘any outcome in particular’, RAND provide ‘as much consolation as justice and impartiality can provide […] to the losers’). For in primis, the proponents of RAND have hitherto failed to provide convincing evidence that individuals generally find it easier to accept unfavourable allocations just because these allocations result from RAND (rather than from DIR). And second, even if individuals found it easier to accept unfavourable allocations just because these allocations result from RAND (rather than from DIR), this would not *per se* license the normative claim that the relevant decision-makers should use RAND (rather than DIR) to allocate SLSR (e.g. the psychological benefits that using RAND rather than DIR putatively yields to the involved individuals do not generally trump the normative relevance of the differences in strength of different individuals’ claims to SLSR and these differences’ life-and-death implications).

6 A proponent of RAND may object that justifying the use of RAND does not require decision-makers to establish that individuals’ claims are equally strong, but only requires them to exclude the presence of obvious reasons or evidence to regard some individuals’ claims as much stronger than others. However, this objection fails to ground a wide-ranging case in favour of RAND unless the proponents of RAND supplement it with plausible and detailed specifications of how much time and resources decision-makers must devote to assessing individuals’ claims to be able to justifiably infer that individuals’ claims are equally strong from the sole fact that they do not discern obvious reasons or evidence to regard some individuals’ claims as much stronger than others. And the proponents of RAND have hitherto failed to address this justificatory challenge (e.g. note no. 10).

7 A proponent of RAND may object that decision-makers’ reliance on DIR may lead them to regard their evaluations of the strength of individuals’ claims to SLSR ‘as far more [reliable] than they are’ (Stone, 2013: 592) and gather information about individuals’ claims beyond the point where the marginal costs of information search offset the marginal benefits of such search (e.g. Stone, 2011: 153). However, decision-makers’ reliance on RAND may have equally problematic effects (e.g. the availability of RAND may lead decision-makers to devote little time and resources to assess the strength of individuals’ claims to SLSR). And as I argue in the coming sections, the relevant decision-makers should often devote more time and resources to assess the strength of individuals’ claims to SLSR than the proponents of RAND maintain .

8 An allocation procedure can be said to give individuals an equal chance of receiving the available SLSR both in the sense that, for all the relevant decision-makers know, it is equally likely that any of these individuals will receive the SLSR (epistemic equiprobability), and in the sense that each of those individuals has equal objective chance of receiving the SLSR (objective equiprobability). In the specialized literature, intense debates have taken place concerning how to distinguish these two notions of equiprobability (e.g. Bradley, 2017) and whether epistemic (as opposed to objective) equiprobability suffices to express one’s commitment to satisfy individuals’ claims equally (e.g. John and Millum, 2020; Hersch and Rowe, 2021). I do not expand on these debates since my response to the argument from equal chances does not directly depend on what position one advocates in such debates.

9 Similar remarks apply if the argument from equal chances is rephrased in terms of expected utility rather than expected benefit (e.g. Singer, 1977). For the notion of expected utility has different normative properties than the notion of utility and one may consistently value equality in utilities without valuing equality in expected utilities (e.g. Broome, 1984a; Fumagalli, 2013, 2019). A proponent of RAND may object that although the chances of receiving SLSR do not provide surrogate satisfaction, giving individuals equal chances often ‘satisfies the demand of fairness to an extent’ (Piller, 2017: 226). I shall address and rebut fairness-related calls for RAND in ‘Argument from Fairness'. For now, I note that giving each of the involved individuals equal chances does not eliminate the inequality inherent in the fact that only some of these individuals receive the SLSR. For the individuals who receive the SLSR receive both the equal chance given to each of the involved individuals and the SLSR itself (e.g. Wasserman, 1996: 44).

10 A proponent of the argument from equal chances may object that decision-makers often lack conclusive reasons/evidence to regard some individuals’ claims to SLSR as stronger than other individuals’ claims to SLSR, and that whenever this is the case decision-makers should treat all the involved individuals’ claims to SLSR as equally strong and use unweighted RAND (e.g. Glover, 1977: 203–227). Yet, decision-makers may simultaneously lack conclusive reasons/evidence to regard some individuals’ claims to SLSR as stronger than other individuals’ claims to SLSR while having convincing reasons/evidence to regard some individuals’ claims to SLSR as stronger than other individuals’ claims to SLSR. Whenever this is the case, using unweighted RAND may lead decision-makers to questionably disregard significant differences between the strength of different individuals’ claims to SLSR (e.g. note no. 6).

11 A proponent of RAND may object that weighted RAND can effectively reduce the risk that individuals with comparatively weaker claims receive SLSR. Yet, on many conceptions of fairness, when the relevant decision-makers can reliably assess the strength of individuals’ claims to the available SLSR, it is unfair to use RAND that frequently deny SLSR to individuals who have comparatively stronger claims to SLSR while giving SLSR to individuals who have comparatively weaker claims to SLSR (e.g. Elster, 1988: 171). Moreover, as argued in the previous sections, weighted RAND face severe difficulties when it comes to apportioning each individual’s chance of receiving the available SLSR to the strength of each individual’s claim to such SLSR.

12 A proponent of the argument from fairness may object that decision-makers may circumvent the difficulties inherent in establishing exactly when the difference between stronger and weaker claims is small enough that RAND are fairer than DIR by adopting ‘a proportional chances scheme […] in the gray area in which it is hard to determine whether a difference is substantial or not’ (Segev, 2005: 250). The idea is to use DIR in cases where distinct individuals’ claims to SLSR significantly differ in strength (e.g. large-difference cases), unweighted RAND in cases where distinct individuals’ claims have exactly (or roughly) equal strength (e.g. small-difference cases), and weighted RAND in all other cases. Regrettably, this proposal remains highly impractical unless it is supplemented with plausible and detailed criteria to demarcate these three types of cases and resolve disagreements about such demarcation issue.

13 The argument from incommensurability takes the strength of different claims to be incommensurable when none of these claims is stronger than the others and the claims are not equally strong either (e.g. Raz, 1986). In recent decades, different views of the relationship between incommensurability and incomparability have been advocated (e.g. Chang, 1997; Broome, 2004; Rabinowicz, 2009). I mention these views in passing since my response to the argument from incommensurability does not directly rest on which of those views one favours.

14 A proponent of RAND may object that when the strength of individuals’ claims to SLSR is hard to assess, many individuals believe that SLSR should be allocated on the basis of RAND (rather than DIR), and therefore the relevant decision-makers should use RAND (rather than DIR). This objection correctly notes that individuals’ views may importantly inform the design and the implementation of allocation procedures (e.g. Biddison *et al.*, 2018, on how community engagement forums informed triage guidelines for influenza pandemic in some US states). However, individuals’ views do not directly determine what allocation procedures should be adopted by the relevant decision-makers (e.g. Savulescu *et al.*, 2019, on cases where strong ethical arguments bear against including the allocation criteria advocated by the involved individuals). Moreover, individuals’ views do not generally favour RAND over DIR (e.g. Wilkinson *et al.*, 2020, for experimental results about a number of hypothetical ventilator withdrawal scenarios).

15 The argument from unjust discrimination targets not just cases where the allocation criteria used by the relevant decision-makers are directly discriminatory (e.g. think of openly racist allocation criteria), but also cases where such criteria are indirectly discriminatory (e.g. Harris, 1995; Rothstein, 2010; Silva, 2020, on how some DIR based on individuals’ medical conditions tend to adversely affect disabled or otherwise disadvantaged individuals because of socio-economic factors that do not directly pertain to these individuals’ medical conditions). I take my response to the argument from unjust discrimination to hold for cases of both direct and indirect discrimination.

16 A proponent of RAND may object that RAND are more effective than DIR in preventing the involved individuals ‘from trying to make themselves more eligible, at cost to themselves or to society’ (Elster, 1988: 166; also Elhauge, 1994, on putative cases where DIR incentivize the involved individuals to withhold information from their physicians). However, both the relevant decision-makers and society at large may value the opportunity to incentivize the involved individuals to adopt morally and socially desirable (e.g. prudent) behaviour. And DIR can often incentivize morally and socially desirable behaviour more effectively and directly than RAND (e.g. Glannon, 1998; Segev, 2005). For on DIR, the strength of individuals’ claims to SLSR directly determines not only what chances different individuals have of receiving the available SLSR, but also which individuals will receive such SLSR (‘Introduction').
